# Potential Association of Reactive Oxygen Species With Male Sterility in Peach

**DOI:** 10.3389/fpls.2021.653256

**Published:** 2021-04-14

**Authors:** Yaming Cai, Zhishen Ma, Collins Otieno Ogutu, Lei Zhao, Liao Liao, Beibei Zheng, Ruoxi Zhang, Lu Wang, Yuepeng Han

**Affiliations:** ^1^CAS Key Laboratory of Plant Germplasm Enhancement and Specialty Agriculture, Wuhan Botanical Garden, The Innovative Academy of Seed Design, Chinese Academy of Sciences, Wuhan, China; ^2^University of Chinese Academy of Sciences, Beijing, China; ^3^Center of Economic Botany, Core Botanical Gardens, Chinese Academy of Sciences, Wuhan, China; ^4^Shijiazhuang Pomology Institute, Hebei Academy of Agricultural and Forestry Sciences, Shijiazhuang, China; ^5^Sino-Africa Joint Research Center, Chinese Academy of Sciences, Wuhan, China

**Keywords:** *Prunus persica*, tapetum degradation, pollen development, ROS homeostasis, male sterility

## Abstract

Male sterility is an important agronomic trait for hybrid vigor utilization and hybrid seed production, but its underlying mechanisms remain to be uncovered. Here, we investigated the mechanisms of male sterility in peach using a combined cytology, physiology, and molecular approach. Cytological features of male sterility include deformed microspores and tapetum cells along with absence of pollen grains. Microspores had smaller nucleus at the mononuclear stage and were compressed into belts and subsequently disappeared in the anther cavity, whereas tapetum cells were swollen and vacuolated, with a delayed degradation to flowering time. Male sterile anthers had an ROS burst and lower levels of major antioxidants, which may cause abnormal development of microspores and tapetum, leading to male sterility in peach. In addition, the male sterility appears to be cytoplasmic in peach, which could be due to sequence variation in the mitochondrial genome. Our results are helpful for further investigation of the genetic mechanisms underlying male sterility in peach.

## Introduction

A typical flower has four types of floral organs: sepal, petal, stamen, and pistil. The function of the flower is to make seeds for plant propagation. Seed development is initiated by fertilization in which male and female gametes fuse, with the former produced in the anther, a part of stamen, whereas the latter is produced in the ovary, an interior part of the pistil. Hence, the development of male and female gametes is crucial for reproduction of flowering plants. However, some plants lack male gametes, also called pollen grains, despite having complete floral organs. The development of pollen grains is a complex and delicate process. Initially, the pollen mother cell in the pollen sac undergoes meiotic division to produce four daughter cells that are called a tetrad. Later, the tetrad dissociates and develops into mononuclear microspores with the nucleus in the center of the cell. Tapetum cells nourish the microspores, increasing in the size and gradually forming a large central vacuole in the cell. As a result, the nucleus gradually moves from the center to the side. Subsequently, the microspores undergo a mitotic division, producing a larger vegetative cell and a smaller generative cell. Most angiosperms, such as lily, cotton, peach, and orange, have bicellular pollen grains, containing vegetative and generative cells, when the pollen is mature. However, some plants, such as cereals and rapeseed, have trinuclear pollen grains as their generative cells undergo additional mitotic division to generate two sperm cells before the pollen is released. Pollen grains at the bicellular and tricellular stages are also referred to as male gametophytes. Previous studies have shown that numerous genes are involved in pollen development (Wilson and Zhang, 2009; Zhang et al., 2011; Gómez et al., 2015). Abnormal structure or expression of pollen development–related genes may cause failure of the anther to produce mature pollen grains, resulting in male sterility (McCormick, 2004; Borg et al., 2009; Cai et al., 2015).

To date, numerous genes that are related to male sterility have been discovered in plants (Singh et al., 2010; Lukaszewski, 2017). Particularly, functional disorder of genes related to tapetum development is one of the main causes of pollen abortion. Tapetum is located in the innermost part of anther tissue, and its main function is to provide nutrition for the development of pollen grains. During the late stages of pollen development, the tapetum undergoes cellular degradation, and anthers subsequently crack, causing mature pollen grains to be released (Mariani et al., 1990; Parish and Li, 2010; Zhu et al., 2011; Xie et al., 2014). Therefore, normal tapetum is crucial for the development of pollen grains (Li et al., 2017). For example, mutations of *OsMS1* and *OsTDR* genes that regulate tapetum development can block the degradation of tapetum, leading to male sterility in rice (Li et al., 2006; Yang et al., 2019).

In addition to the dysfunction of genes involved in the regulation of tapetum development, other factors such as the excessive production of reactive oxygen species (ROS) in anthers can also cause male sterility. For example, reducing the expression of *MT-1-4b* gene encoding a type 1 small Cys-rich and metal binding protein increases the level of superoxide anion (O_2_^–^), which affects tapetum development and thus decreases pollen infertility (Hu et al., 2011). ROS can act as signaling molecules and maintaining their basal levels is essential for plant development (Gechev et al., 2006). Mitochondrion is a main source of ROS production as it is the site of oxidative phosphorylation. Mitochondria structural variation can lead to the ROS burst (Shadel and Horvath, 2015). In addition, the ROS burst can be induced by environmental stress and dysfunction of genes associated with ROS elimination (Gechev et al., 2006; Suzuki et al., 2012; Miller et al., 2010). Plants contain a variety of antioxidants, such as carotenoids, glutathione (GSH), ascorbate, and flavonoids, which serve as ROS scavengers (Mittler et al., 2004). In plants, the production and elimination of ROS are a balanced process, which is called ROS homeostasis. Many studies have shown that loss of ROS homeostasis can lead to male sterility (Hu et al., 2011; Qu et al., 2014; Zhou et al., 2019; Wang et al., 2020; Zhu et al., 2020).

Peach (*Prunus persica*) is a fruit tree widely cultivated in temperate regions of the world, with ornamental and edible properties. Although male sterile frequently occurs in peach, its molecular mechanism is yet to be fully uncovered. In this study, we investigated the mechanism of male sterility in “Jinxiang” (“JX”), a popular yellow flesh peach cultivar in China. Our results indicated that abnormal development of tapetum and microspores are caused by disruption of ROS homeostasis, resulting in male sterility. This finding provides an insight into mechanisms underlying male sterility in peach.

## Materials and Methods

### Plant Materials

Peach varieties used in this study are maintained in the orchard at Shijiazhuang Pomology Institute, Hebei Academy of Agriculture and Forestry Sciences, Shijiazhuang, China. Anthers were collected from a male sterile cultivar, JX, and a fertile cultivar, “Zaohong” (“ZH”), as a control. These two cultivars have the same flowering time. Samples were collected at 5-day intervals from March 09, 2019, to April 03, 2019. All samples were divided into six stages based on sampling time, S0, S1, S2, S3, S4, and S5, which were confirmed by paraffin section according to previously reported protocol (Fang et al., 2016). S0–S3 corresponded to 20, 15, 10, and 5 days before anthesis, respectively. S4 corresponded to the anthesis, and S5 represented 5 days after anthesis.

### Anatomical Analysis of the Anther Structure

Anthers from the unopened and fully opened flower buds were taken out, put in culture dish, and left to dry. The dried anthers were fixed with glutaraldehyde (pH 6.8) at 4°C for 1.5 h and then washed three times with 0.1 mol/L phosphoric acid buffer (pH 6.8) for 10 min each. Subsequently, the treated samples were dehydrated with 50, 70, 80, and 90% ethanol for 10–15 min each time and finally dehydrated three times with 100% ethanol for 10–15 min each time. Then, the anthers were immersed in 100% ethanol:tert-butanol (vol/vol = 1) for 15 min and transferred to pure tert-butanol for 15 min. The treated samples were dried for 4 h using LABCONCO Freeze Vacuum Dryer (Labconco, MO, United States). Finally, the observation surface of the sample was put in an upward position, fixed with conductive tape, and sprayed with gold powder using Leica EM ACE Ion sputtering coating instrument (Leica, Wetzlar, Germany). Anther morphology was observed using scanning electron microscope (SEM) (Hitachi, Tokyo, Japan). In addition, transmission electron microscopy (TEM) was performed according to a previous report (Li et al., 2006).

### Detection of Pollen Viability

Pollen viability was analyzed by both 1% iodine/potassium iodide (I_2_/KI) and fluorescein diacetate (FDA) staining. For the I_2_/KI assay, the samples were stained for 5 min and scanned with microscope (Nikon, Tokyo, Japan). For the FDA staining assay, the pollen grains were immersed in a concave slide containing 2 μg/mL FDA solution for 30 min and observed under the fluorescence microscope (Nikon, Tokyo, Japan) after washing out FDA.

### Measurement of ROS and Antioxidant Contents in Anthers

The hydrogen peroxide (H_2_O_2_) content in anthers was measured using H_2_O_2_ assay kit (Beyotime, Shanghai, China) following the manufacturer’s instruction. The O_2_^–^ radical content in anthers at each developmental stage was assayed using nitro-blue tetrazolium (NBT) staining according to a previous report (Xie et al., 2014).

The content of GSH was measured using GSH assay kit (Beyotime, Shanghai, China) according to the manufacturer’s instruction. Carotenoid content was measured using plant carotenoid detection kit (Solarbio, Beijing, China) following the manufacturer’s instruction. The content of ascorbate peroxidase (APX) and GSH *S*-transferase (GST) was measured by APX and GST detection kit (GeRuiSi, Nanjing, China), respectively, according to the manufacturer’s instruction.

### RNA-Sequencing Analysis

Three development stages of JX and ZH anthers, S0, S1, and S3, were selected for RNA-sequencing (RNA-seq) analysis. RNA extraction and purity, library construction, and RNA-seq were conducted according our previous report (Wang L. et al., 2013). After removing adapters and low-quality reads, clean reads were mapped to the peach reference genome (International Peach Genome Initiative, 2013). Gene expression levels were estimated based on the value of expected number of fragments per kilobase of transcript sequence per million base pairs sequenced (FPKM). Differentially expressed genes (DEGs) were identified using the following criteria: fold change ≥2.0 and a false discovery rate <0.01.

### RNA Extraction and Quantitative Reverse Transcription–Polymerase Chain Reaction Analysis

Total RNA extraction was performed using Plant RNA Extraction Kit (Aidlab Biotech, Beijing, China), and reverse transcription was conducted using Reverse Transcriptase Kit (M-MLV) (Zomanbio, Beijing, China) according to the manufacturer’s instructions. Quantitative reverse transcription–polymerase chain reaction (qRT-PCR) was performed using TAKARA SYBR^®^ Premix EX Taq TM II (Tli RNaseH Plus), and the amplification program was as follows: one cycle of 30 s at 95°C, followed by 40 cycles of 5 s at 95°C and 34 s at 60°C. Relative expression levels were normalized against the reference gene *GADPH* (Tong et al., 2009). Each treatment contained three biological replicates. Primer sequences for qRT-PCR are listed in Supplementary Table 1.

## Results

### JX Has Abnormal Anther Morphology and Lacks Pollen Grains

**FIGURE 1:**
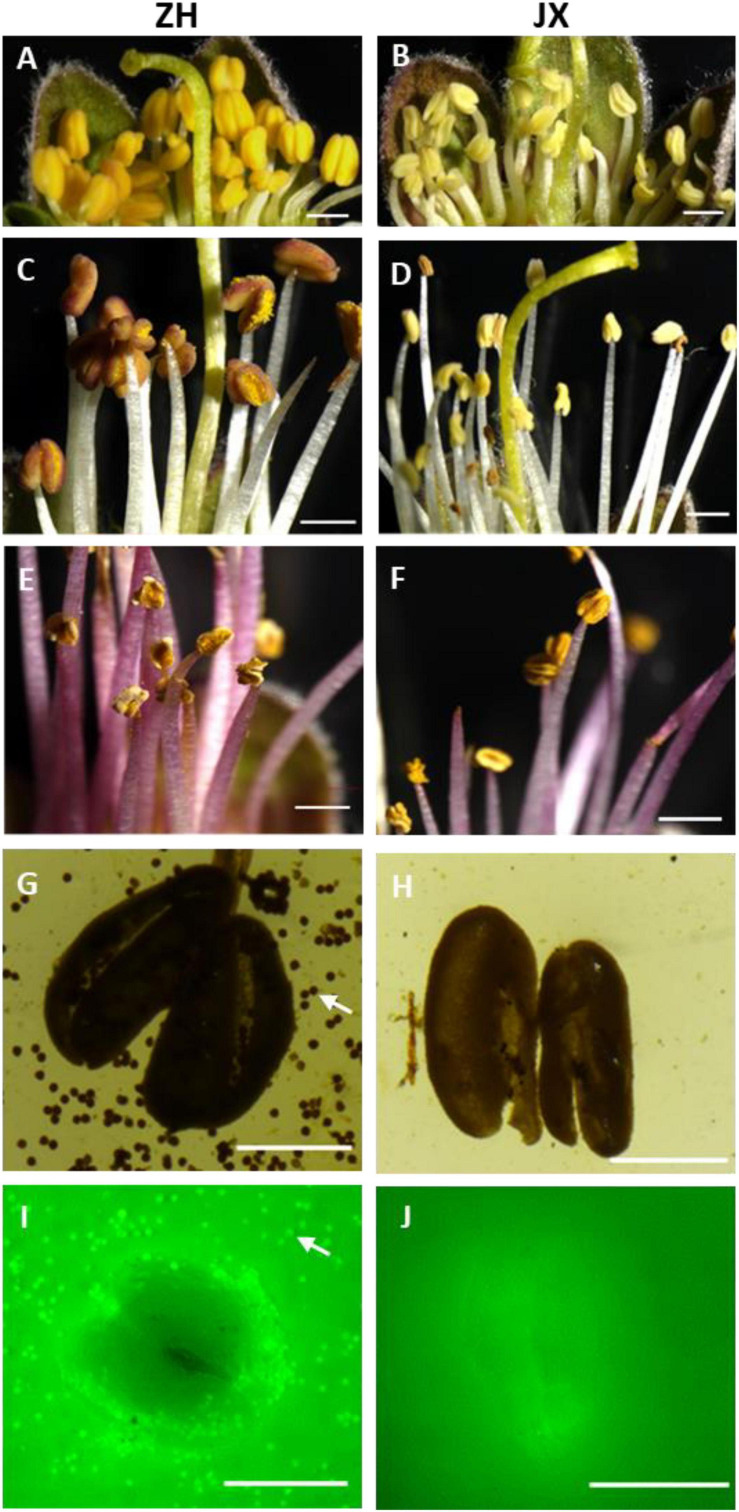
Comparison of anther phenotype between “ZH” and “JX”. Panels **(A,B)** show swollen and shrunken anthers in flower buds, respectively. **(C,D)** Anthers at the anthesis. **(E,F)** Anthers 5 days after flowering. **(G,H)** Cracked anthers along with pollen grains stained using I_2_/KI. Panels **(I,J)** cracked anthers and pollen grains stained using FDA. The white arrow indicates pollen grains. The white bar represents 1,000 μm in **(A–F)**, and 500 μm in **(G–J)**.

Flower buds showed no obvious difference in appearance between the male sterile cv. JX and the fertile cv. ZH before the full blooming stage (Supplementary Figure 1), but their inner anthers were different in size and color (Figures 1A,B). The anthers of ZH were yellow, swollen, and significantly larger than the shrunken anthers of JX that were pale in color. The anthers of ZH cracked to release pollen grains, and the color changed from yellow to dark orange at the anthesis, whereas the anthers of JX were indehiscent with no obvious change in color (Figures 1C,D). Five days after anthesis, the anthers of ZH wilted, whereas dehiscence was observed in some anthers of JX, with filament color both changing to purple (Figures 1E,F). Both I_2_/KI and FDA staining showed that the anthers of ZH contained viable pollen grains, whereas the anthers of JX had no pollen grains (Figures 1G–J).

**FIGURE 2:**
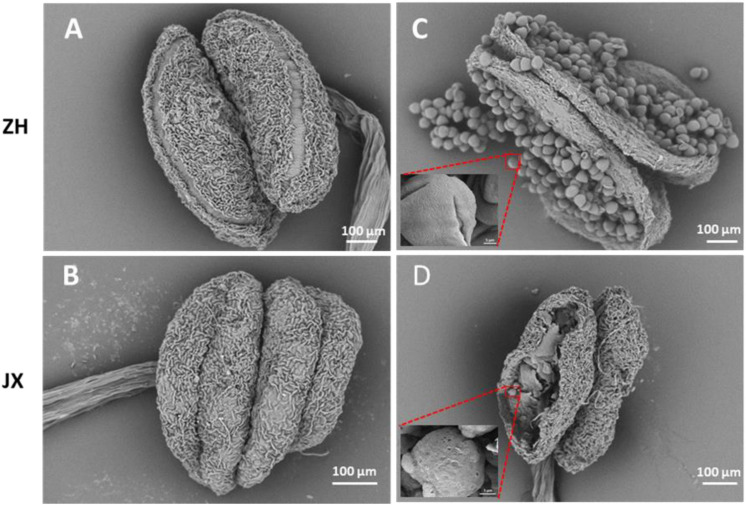
Comparison of anther morphology between “ZH” and “JX” using SEM. **(A,B)** Anthers before dehiscence. **(C,D)** Anthers after dehiscence.

Scanning electron microscope assay showed that the surface of ZH anthers had opening cracks, whereas abnormal opening cracks were observed in the surface of JX anthers (Figures 2A,B). The anthers of ZH released numerous pollen grains during dehiscence, whereas the disrupted anthers of JX had no pollen grains, with few pollen-like spheres (Figure 2D) that had no typical wrinkles on their surface as shown in Figure 2C.

In summary, the above results suggested that the anthers of JX underwent abnormal development, with delayed dehiscence in some anthers that had no pollen grains.

### Microspores of JX Were Compressed Into Bands That Disappeared in Anther Cavity

**FIGURE 3:**
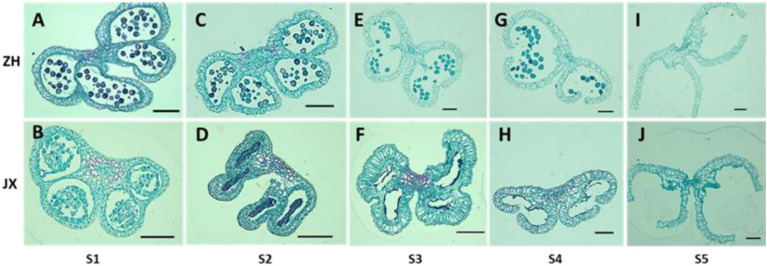
Comparison of transverse section of anthers between “ZH” and “JX” at five development stages as described in Section “Materials and Methods.” All bars represent 100 μm. **(A,C,E,G,I)** Transverse section of ‘ZH’ anthers. **(B,D,F,H,J)** Transverse section of ‘JX’ anthers.

To investigate the cytological mechanism of pollen abortion in JX, we examined paraffin-embedded anther samples at five stages (S1–S5) using microscope. Microspores were abundant in the anthers of both ZH and JX at S1; however, the anthers JX showed irregular morphology (Figures 3A,B). Microspores in each pollen sac were compressed into a single band in JX, whereas no morphological change was detected for microspores of ZH (Figures 3C,D). The anthers of ZH cracked normally during the S3 stage, but not for the anthers of JX that had empty pollen sacs, with no pollen grains (Figures 3E,F). Interestingly, anther cracking was delayed to the anthesis in JX (Figure 3H), in which mature pollen grains were released in ZH (Figure 3G). At S5, anthers showed no difference in structure between ZH and JX (Figures 3I,J). These results suggested that microspore development was disrupted, leading to pollen abortion in JX.

### Ultrastructural Feature of Microspores and Tapetum in ZH and JX

**FIGURE 4:**
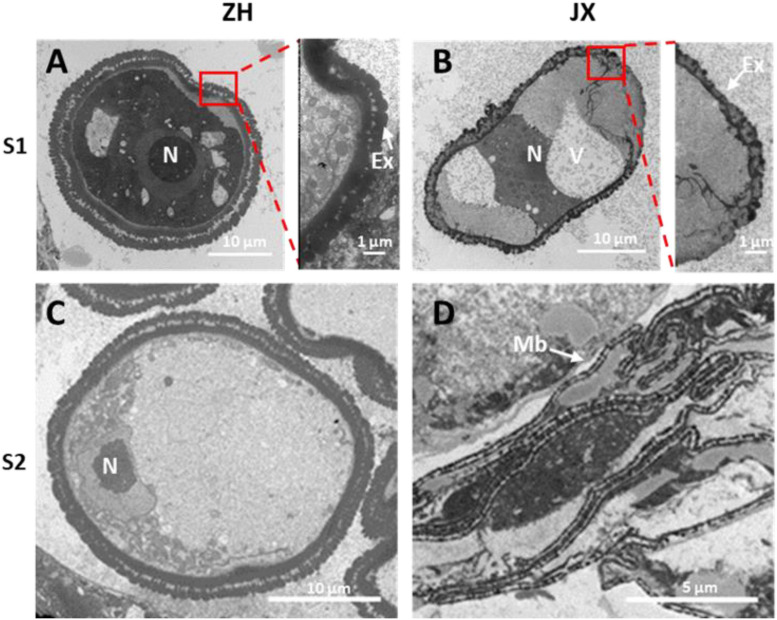
Transmission electron microscopy (TEM) scans of microspore features at two stages in “JX” and “ZH.” N, nucleus; Ex, exine; V, vacuole; Mb, microspore bands. **(A,C)** represent microspores of ‘ZH’ at S1 and S2, respectively. **(B,D)** indicate microspores of ‘JX’ at S1 and S2, respectively.

To gain deep insights into abnormal anther development in JX, the ultrastructures of microspores and tapetum were examined using TEM. A single nucleus was present in microspores at S1 and S2 in JX and ZH (Figure 4). The microspores of ZH contained large nucleus, whereas the microspores of JX were severely vacuolated, with smaller nucleus (Figures 4A,B). Moreover, the exine of microspores in ZH had regular rod-like protrusions, whereas the exine structure of microspores in JX was irregular and incomplete (Figures 4A,B). At S2, the nuclear in microspores of ZH was pushed to the side because of vacuole enlargement, whereas microspores of JX were compressed into bands (Figures 4C,D).

**FIGURE 5:**
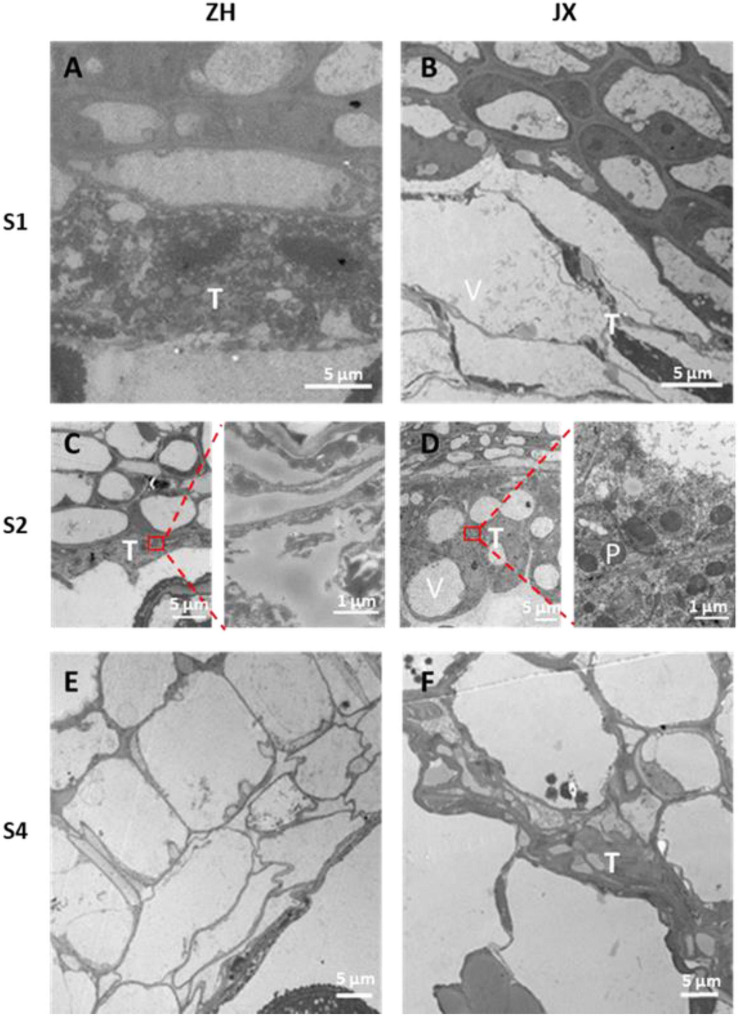
Transmission electron microscopy scans of tapetum morphology in “ZH” and “JX.” **(A)** Normal tapetum; **(B)** vacuolized tapetum; **(C)** degraded tapetum; **(D)** swollen tapetum; **(E)** completely degraded tapetum; **(F)** delayed tapetum degradation. T, tapetum; V, vacuole; P, peroxisome.

Tapetum degradation occurred at S1 in ZH, whereas the development of tapetum was abnormal in JX (Figures 5A,B). The tapetum cells of JX had loose cytoplasm and heavy vacuolization in contrast to those of ZH at S1. The tapetum was almost completely degraded at S2 in ZH (Figure 5C), whereas the tapetum cells of JX were swollen and contained large vacuoles (Figure 5D). At S4, the tapetum of ZH was completely degraded (Figure 5E), whereas tapetum degradation was just initiated in JX (Figure 5F). Altogether, these results suggested that tapetum development was abnormal and could not be timely degraded to nourish microspores, leading to pollen abortion in JX. In addition, we observed that tapetal cells at S2 were filled with round, electron dense, and granular organelles, which are typical characteristics of peroxisomes (Figure 5D). This suggested the presence of oxidative stress in microspores of JX (Hu et al., 2011).

### The ROS Content Was Elevated in the Anthers of JX Compared to Those of ZH

**FIGURE 6:**
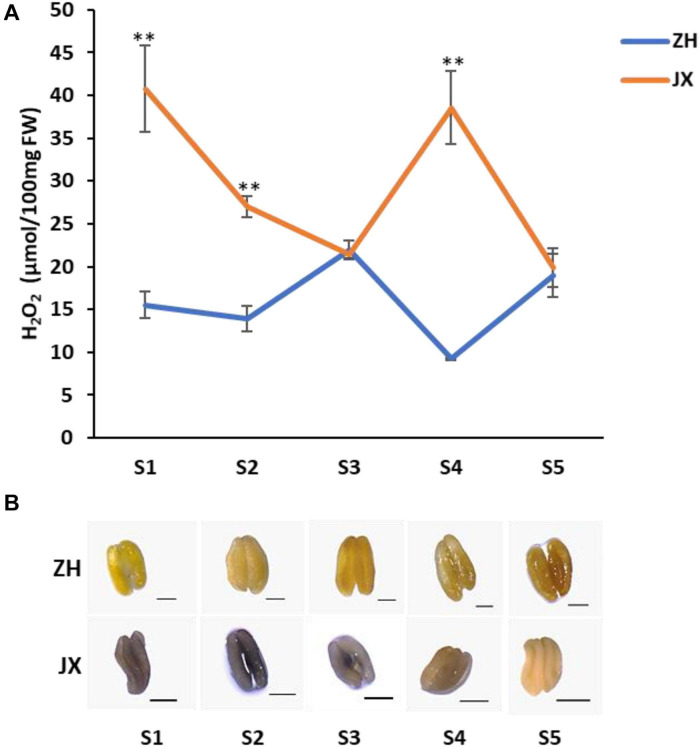
The concentration of ROS in anthers of “ZH” and “JX” throughout their development. **(A)** Accumulation of H_2_O_2_ in anthers at stages 1–5. Error bars indicate standard deviation (SD) of three biological replicates. Statically significant differences at *P* ≤ 0.01 (Student *t*-test) is indicated by ^∗∗^. **(B)** Measurement of O_2_^–^ accumulation in anthers at stages 1–5 using NBT staining. The intensity of blue color indicates high content of O_2_^–^. All bars represent 500 μm.

As oxidative stress is associated with excess production of ROS that causes damage to cell structure (Gechev et al., 2006), we measured the contents of H_2_O_2_ and O_2_^–^, two main components of ROS (Apel and Hirt, 2004), in anthers of JX and ZH at stages 1–5. The concentration of H_2_O_2_ was significantly higher in JX than in ZH at S1, S2, and S4 (Figure 6A). However, the concentration of H_2_O_2_ in anthers at S3 and S5 was similar between JX and ZH. Moreover, the NBT staining assay demonstrated that the content of O_2_^–^ at stages 1–4 was higher in JX than in ZH (Figure 6B). These results suggested an excessive accumulation of ROS in the anthers of JX.

### Comparative Transcriptome Analysis Between the Anthers of JX and ZH

As mentioned previously, abnormal development of microspores was initially detected at S1. Thus, S0 and S1 represent transitional stages that are crucial for comparative transcriptome analysis according to a previous study (Liao et al., 2020). Moreover, microspores gradually degraded and finally disappeared in S3. Based on these findings, three stages, S0, S1, and S3, were selected to conduct comparative transcriptome analysis to further reveal molecular mechanisms of pollen abortion in JX. A total of 18 libraries were sequenced, generating approximately 5.77-Gb raw reads for each library, with an average Q30 value of 94.77% (Table 1). The clean reads of each sample were mapped against the peach reference genome^1^, with unique mapping rates ranging from 81.64 to 92.57%. Pearson correlation coefficients were greater than 0.9 among three biological replications of each sample (Supplementary Figure 2), and 3D principal component analysis (PCA) plot displayed that three biological repeats of each sample clustered together (Supplementary Figure 3). This indicated high consistency between biological replicates, which is suitable for conducting comparative transcriptome analysis.

**TABLE 1:** Summary of RNA-seq data for anthers of two peach cultivars at three stages*.

**Library**	**Total reads**	**Clean reads**	**Clean base (bp)**	**Mapped reads**		**Unique mapped reads**		**GC content**	**% ≥ Q30**
				**No.**	**Percentage**	**No.**	**Percentage**		
B1-S0	55,299,248	27,649,624	8,241,443,970	52,624,756	95.16%	51,189,336	92.57%	46.36%	95.46%
B2-S0	52,472,066	26,236,033	7,835,470,872	49,381,068	94.11%	47,921,503	91.33%	46.38%	94.58%
B3-S0	42,518,758	21,259,379	6,319,171,812	39,177,432	92.14%	38,093,507	89.59%	46.78%	95.09%
B1-S1	49,545,076	24,772,538	7,394,915,150	45,810,911	92.46%	43,410,563	87.62%	46.58%	94.70%
B2-S1	43,014,382	21,507,191	6,426,544,328	39,549,654	91.95%	37,331,283	86.79%	46.63%	95.10%
B3-S1	44,495,164	22,247,582	6,633,928,720	41,781,723	93.90%	39,363,989	88.47%	46.48%	94.57%
B1-S3	51,072,856	25,536,428	7,617,437,418	48,634,923	95.23%	46,403,165	90.86%	46.56%	94.67%
B2-S3	55,679,980	27,839,990	8,304,340,272	53,371,184	95.85%	51,077,734	91.73%	46.32%	94.87%
B3-S3	46,058,244	23,029,122	6,861,185,988	43,908,560	95.33%	42,138,758	91.49%	46.36%	94.99%
C1-S0	39,985,662	19,992,831	5,954,533,580	36,333,712	90.87%	35,370,832	88.46%	46.29%	94.36%
C2-S0	41,941,794	20,970,897	6,239,724,192	38,482,650	91.75%	37,116,815	88.50%	46.54%	94.96%
C3-S0	49,636,558	24,818,279	7,395,890,042	46,277,979	93.23%	44,850,103	90.36%	46.26%	95.02%
C1-S1	40,903,146	20,451,573	6,112,103,674	37,830,395	92.49%	36,339,637	88.84%	46.51%	94.36%
C2-S1	50,895,486	25,447,743	7,586,570,536	46,982,994	92.31%	45,503,392	89.41%	46.31%	95.26%
C3-S1	45,892,436	22,946,218	6,841,578,052	38,945,963	84.86%	37,464,526	81.64%	48.18%	94.58%
C1-S3	42,742,096	21,371,048	6,378,901,298	38,510,897	90.10%	36,854,266	86.22%	46.95%	94.41%
C2-S3	38,611,318	19,305,659	5,767,550,644	34,646,264	89.73%	33,158,174	85.88%	46.82%	94.31%
C3-S3	41,971,228	20,985,614	6,270,251,464	38,802,892	92.45%	37,066,309	88.31%	46.60%	94.65%

*B and C represent “ZH” and “JX”, respectively. S0, S1, and S3 indicate three developmental stages of anthers. Each sample contains three biological replicates.

**FIGURE 7:**
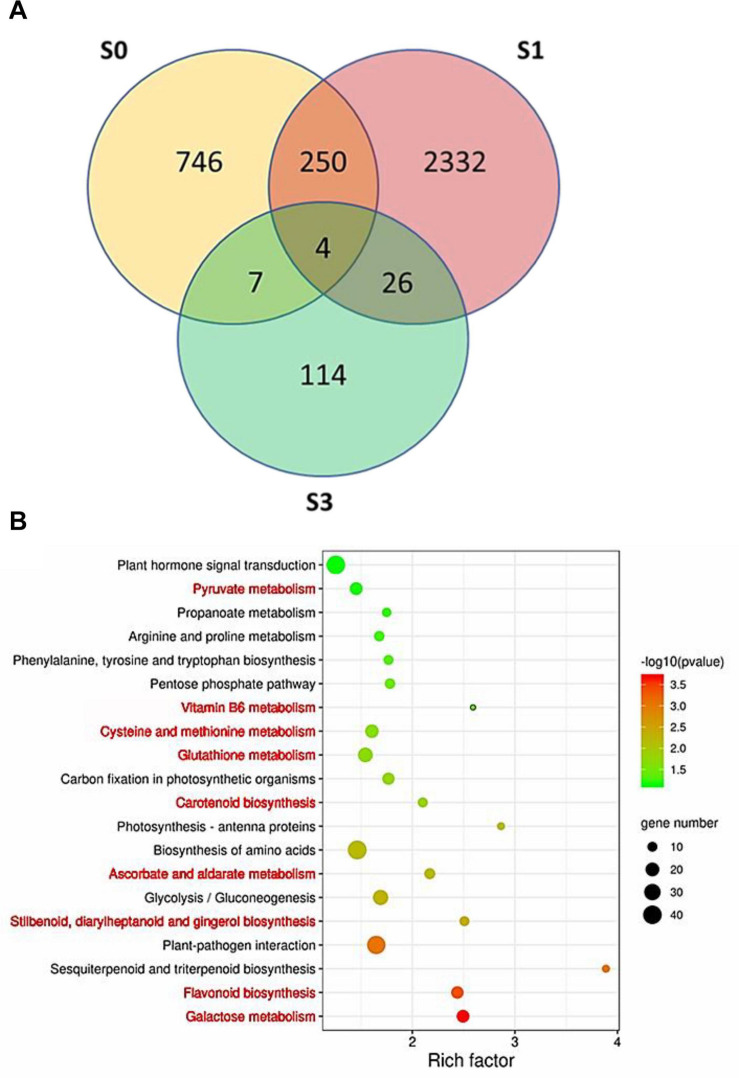
Identification of differentially expressed genes (DEGs) between the anthers of “JX” and “ZH.” **(A)** Venn diagram of DEGs at S0, S1, and S3. **(B)** Top 20 most represented categories of KEGG and the gene number predicted to belong to each category. The pathways involved in ROS homeostasis system are highlighted in red color.

A total of 1,007, 2,612, and 151 DEGs were identified between ZH and JX at S0, S1, and S3, respectively (Figure 7A). Gene Ontology (GO) analysis indicated that the all DEGs could be classified into three types: biological process, cellular components, and molecular function (Supplementary Figure 4). The top 20 most enriched pathways in Kyoto Encyclopedia of Genes and Genomes (KEGG) analysis are shown in Figure 7B. Nine of the 20 enriched pathways are associated with ROS removal processes: (1) galactose metabolism (Hafsa et al., 2019); (2) flavonoid biosynthesis (Agati et al., 2012; Xie et al., 2015; Wei et al., 2019); (3) stilbenoid (Nassiri-Asl and Hosseinzadeh, 2016; Treml et al., 2019), diarylheptanoid (Llano et al., 2019), and gingerol biosynthesis (Dugasani et al., 2010; Si et al., 2018); (4) ascorbate and aldarate metabolism (Zechmann, 2018); (5) carotenoid biosynthesis (Kljak and Grbeša, 2015; Milani et al., 2017; Rojas-Garbanzo et al., 2017); (6) GSH metabolism (Noctor et al., 2012); (7) cysteine (Preczenhak et al., 2019) and methionine metabolism (Bender et al., 2008); (8) vitamin B_6_ metabolism (Chandrasekaran and Chun, 2018); and (9) pyruvate metabolism (Ramos-Ibeas et al., 2017; Guarino et al., 2019). The DEGs involved in these ROS-related pathways are listed in Supplementary Table 2. These ROS-related DEGs might be responsible for the difference in the ROS content between the anthers of ZH and JX.

### The Content of Major Antioxidants in the Anthers Was Lower in JX Than in ZH

**FIGURE 8:**
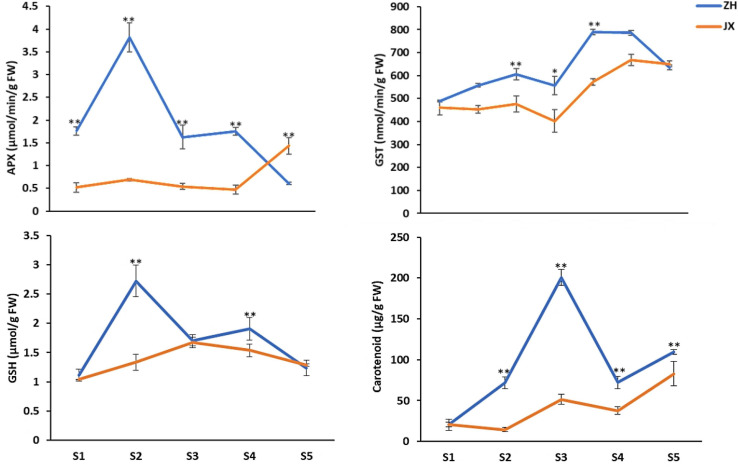
Content of antioxidants in the anthers of “ZH” and “JX” at stages 1–5. Error bars indicate ±SD of three biological replicates. Statically significant differences at *P* ≤ 0.05 and *P* ≤ 0.01 (Student *t* test) are indicated by ^∗^ and ^∗∗^, respectively.

To further confirm the association of pollen abortion with the ROS metabolism in JX, we measured the content of four major ROS scavengers in the anthers at S1–S5. Overall, the concentrations of APX and GST were significantly lower in anthers of JX than in those of ZH throughout the development (Figure 8). Similarly, the contents of carotenoids and GSH were significantly lower in the anthers of JX than in those of ZH. These results indicated there is a disruption of ROS homeostasis in anthers of JX.

**FIGURE 9:**
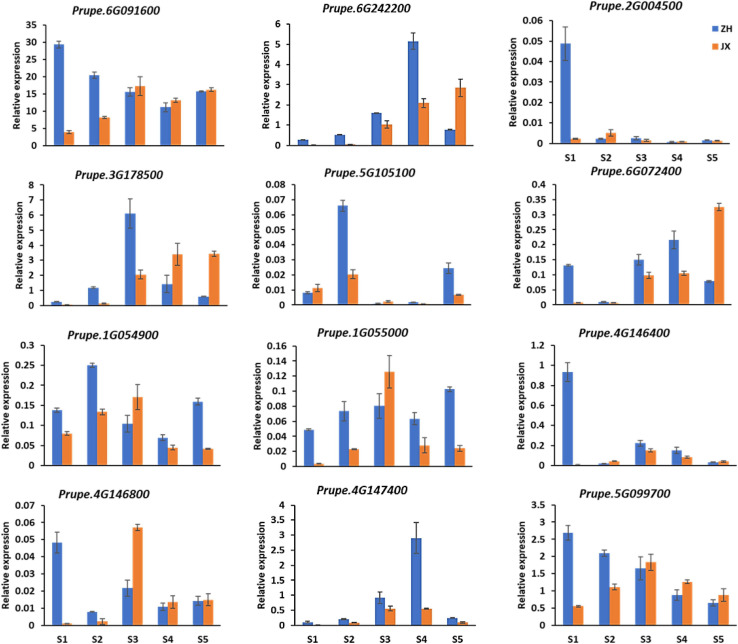
Expression analysis of DEGs by qRT-PCR at S1–S5. Error bars indicate standard deviation from three biological and technical replicates of qRT-PCR.

Screening the DEGs mentioned previously revealed 12 DEGs involved in the biosynthesis of the four major antioxidants, including five (*Prupe.1G054900*, *Prupe.1G055000*, *Prupe.4G146400*, *Prupe.4G146800*, and *Prupe.4G147400*) encoding GST, one (*Prupe.5G099700*) encoding glutamate–cysteine ligase, two (*Prupe.6G091600* and *Prupe.6G242200*) encoding L-APX proteins, one (*Prupe.2G004500*) encoding zeaxanthin epoxidase, one (*Prupe.3G178500*) encoding phytoene synthase, one (*Prupe.6G072400*) encoding cytochrome P450m, and one (*Prupe.5G105100*) encoding beta-carotene hydroxylase. qRT-PCR showed that these genes had lower levels of expression in the anthers of JX at early stages than in the anthers of ZH (Figure 9), which is consistent with the observed lower content of major antioxidants in JX compared with ZH.

## Discussion

### Pollen Abortion Is Associated With Abnormal Development of Microspores and Tapetum in Peach

To our knowledge, this study reports for the first time the cytological and physiological traits associated with male sterility in peach. Our results show that microspores were severely vacuolated, with deformed and incomplete exine structure, and compressed into belts that disappeared at mononuclear stage in male sterile cv. JX. Moreover, the tapetum cells were swollen, vacuolated, with a delayed degradation to flowering time. Thus, male sterility in JX is characterized by abnormal development of microspores and tapetum at mononuclear stage of pollen development. As mentioned previously, tapetum provides nutrition for the development of pollen grains. Abnormal development of microspores could be partially attributed to the disrupted development of tapetum in peach.

Similar phenotype of male sterility has also been reported in model plants such as rice and *Arabidopsis*, in which pollen abortion is frequently found to be associated with genes that are involved in tapetum development and degradation. For example, mutations of tapetum development-related genes in *Arabidopsis*, such as *MYB103*, *MS1*, *TDF*, *DYT1*, *PRX9*, and *PRX10*, can lead to comparable pollen abortion phenotypes to those observed in this study (Ito et al., 2007; Zhang et al., 2007; Zhu et al., 2008, 2015; Jacobowitz et al., 2019). In rice, functional mutations of *TDR*, *API5*, *DEX1*, *GPAT3*, and *DPW3* cause delayed degradation of tapetum, leading to male sterility with features similar to those in this study (Li et al., 2006, 2011; Yu et al., 2016; Men et al., 2017; Mondol et al., 2020). Therefore, degradation of tapetum in mononuclear stage is crucial for normal development of microspores into mature pollen grains. The delayed degradation of tapetume is likely responsible for male sterility in peach cv. JX.

### Disruption of ROS Homeostasis May Hinder Pollen Development, Leading to Male Sterility in Peach

In plants, basal concentration of ROS is important in maintaining normal growth and development. The production and elimination of ROS are a complex network involving participation of multiple factors (Gechev et al., 2006; Miller et al., 2010; Suzuki et al., 2012; Shadel and Horvath, 2015). In this study, the anthers of JX were found to accumulate higher levels of H_2_O_2_ and O_2_^–^ at early stages of development. ROS burst in anther has been found to affect tapetum degradation time (Jiang et al., 2007; Huang et al., 2012; Luo et al., 2013; Wang K. et al., 2013; Yan et al., 2014). ROS burst at the mononuclear stage is likely responsible for the delay of tapetum degradation, leading to abortion of microspores in JX. This finding is consistent with previous reports that disruption of ROS homeostasis contributes to male sterility. For example, the *BZR1* gene is involved in ROS production, and its mutation can delay tapetal cell degeneration, leading to male sterility in tomato (Yan et al., 2020), whereas increased ROS content negatively impacts pollen development in rice (Qu et al., 2014; Zheng et al., 2019; Guo et al., 2020; Zafar et al., 2020).

Plants have a complex enzymatic and non-enzymatic antioxidant system for maintaining the ROS homeostasis (Mittler et al., 2004). In this study, both major enzymatic antioxidants, APX and GST, and major non-enzymatic antioxidants, GSH and carotenoids, were detected in the anthers of ZH and JX. However, the levels of enzymatic antioxidants and non-enzymatic antioxidants were significantly lower in the anthers of JX than in those of ZH. Thus, ROS homeostasis seems to be crucial for pollen development, and its disruption could be associated with male sterility in peach. In addition, a large number of structural genes related to antioxidant production were differentially expressed in the anthers of ZH and JX. It is worthy of further studies to investigate whether antioxidant accumulation is controlled by regulatory genes in peach.

Mitochondrion is a major site of ROS production that is related to oxidative phosphorylation (Gechev et al., 2006; Miller et al., 2010; Suzuki et al., 2012; Shadel and Horvath, 2015). Dysfunction of mitochondrial genes has been reported to cause excessive production of ROS (Touzet and Meyer, 2014). Insertion of unknown open reading frames into the mitochondrial genome causes excessive accumulation of ROS, leading to male sterility in rice (Luo et al., 2013; Wang K. et al., 2013; Xie et al., 2018). In this study, cytological and physiological characteristics of male sterility in JX were similar to those of cytoplasmic male sterile rice variety, Honglian. In addition, ROS burst was observed in the anther of JX, along with six mitochondrial genes involved in oxidative phosphorylation that were differentially expressed in the anthers of ZH and JX (Supplementary Table 3). These findings suggest that male sterility could be cytoplasmic due to sequence variation in the mitochondrial genome in peach, which is consistent with previous finding that ROS burst during pollen development causes Honglian type cytoplasmic male sterility (CMS-HL) in rice (Wang K. et al., 2013). Analysis of structural variation of mitochondrial genome will be a practical way to identify potential candidate genes for male sterility in peach. Notably, a previous study has reported a candidate gene on chromosome 6 (*Prupe.6G024900*) controlling male sterility in peach (Eduardo et al., 2020). However, based on RNA-seq, *Prupe.6G024900* showed no expression in the anther at S0, S1, and S3 of both JX and ZH, suggesting it is unlikely responsible for male sterility in JX. More studies are needed to clarify whether diverse mechanisms are associated with male sterility in peach germplasm.

In summary, our study reveals that disruption of ROS homeostasis may cause abnormal development of microspores and tapetum, leading to cytoplasmic-type male sterility in peach. Our results will be useful for further investigation of mechanisms underlying male sterility in peach.

## Data Availability Statement

The original contributions generated for this study are included in the article/Supplementary Material, further inquiries can be directed to the corresponding author.

## Author Contributions

YC conducted most experiments of this study and wrote the manuscript. YC, ZM, and LZ prepared the experimental materials. LL, LW, and BZ participated in the transcriptomic analysis. YH was overall project leader and revised the manuscript. CO and RZ revised the manuscript. All authors read and approved the final manuscript.

## Conflict of Interest

The authors declare that the research was conducted in the absence of any commercial or financial relationships that could be construed as a potential conflict of interest.

## Footnotes

1 https://www.rosaceae.org/species/prunus_persica/genome_v2.0.a1

## Supplementary Material

Supplementary Figure 1 Appearance of flower buds at different developmental stages in “ZH” and “JX.” Bar = 1 cm.

Supplementary Figure 2 The Pearson correlation between samples. B represents “ZH,” and C represents “JX.”

Supplementary Figure 3 The 3D PCA plot between samples. B represents “ZH,” and C represents “JX.”

Supplementary Figure 4 GO classifications of DEGs at S0, S1, and S3.

Supplementary Table 1 The primers used in this article.

Supplementary Table 2 Differentially expressed genes (DEGs) involved in ROS-related pathways and their description.

Supplementary Table 3 Mitochondrial DEGs involved in oxidative phosphorylation and their description.
