# Supplementary material for: Potential Association of Reactive Oxygen Species With Male Sterility in Peach
Source: Front Plant Sci. 2021 Apr 14;12:653256. doi: 10.3389/fpls.2021.653256 (PMC8079786; doi:10.3389/fpls.2021.653256)
Supplement: Supplementary Figure 1 — Appearance of flower buds at different developmental stages in “ZH” and “JX.” Bar = 1 cm. [file Data_Sheet_1.docx]

**
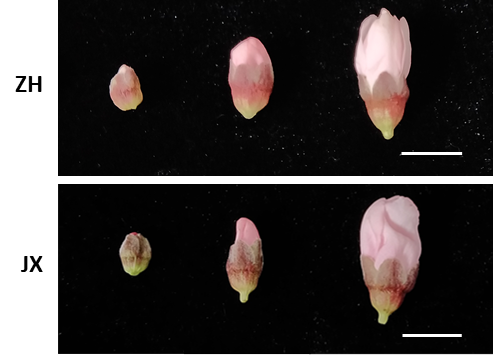
**

**Fig. S1**. Appearance of flower buds at different developmental stages in ‘ZH’ and ‘JX’. Bar = 1 cm.


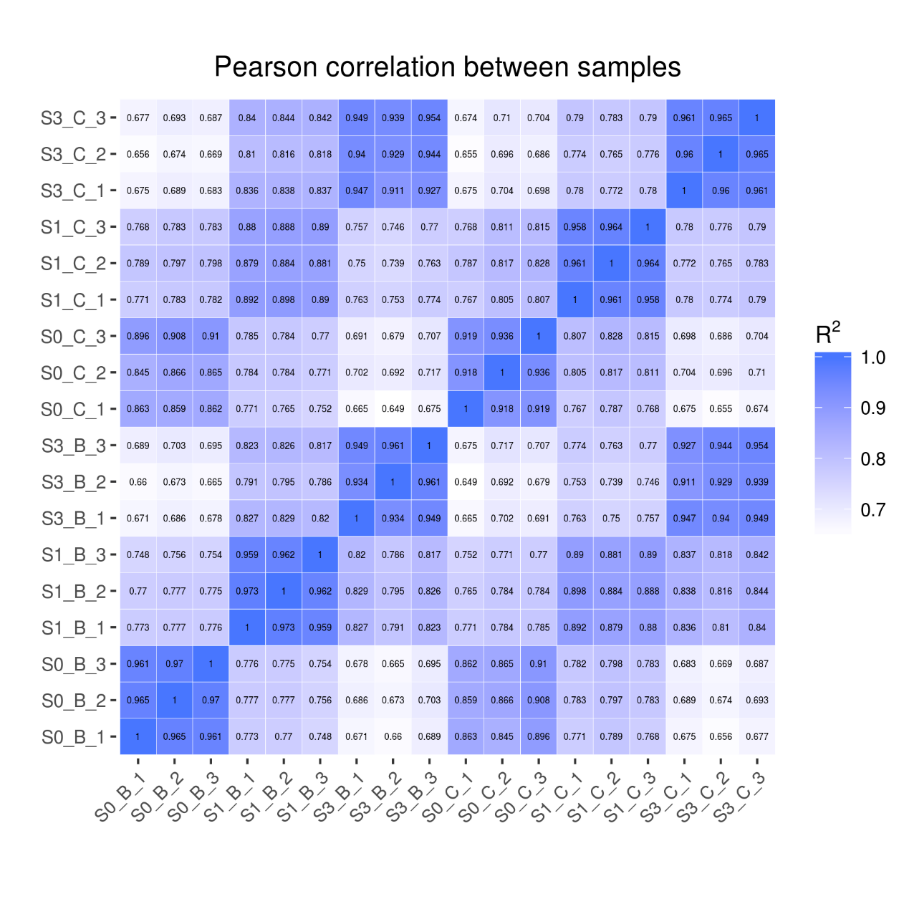


**Fig. S2.** The Pearson correlation between samples. B represents ‘ZH’, C represents ‘JX’.


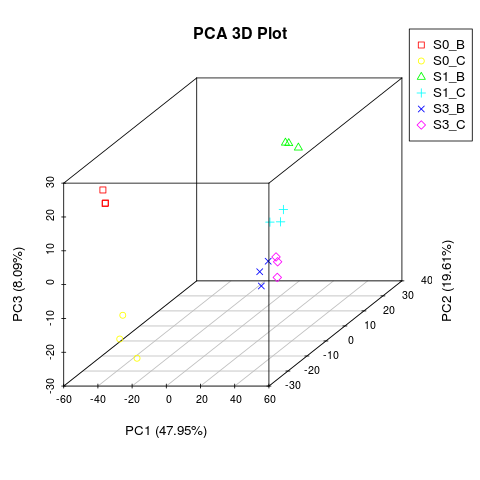


**Fig. S3.** The 3D PCA plot between samples. B represents ‘ZH’, C represents ‘JX’.


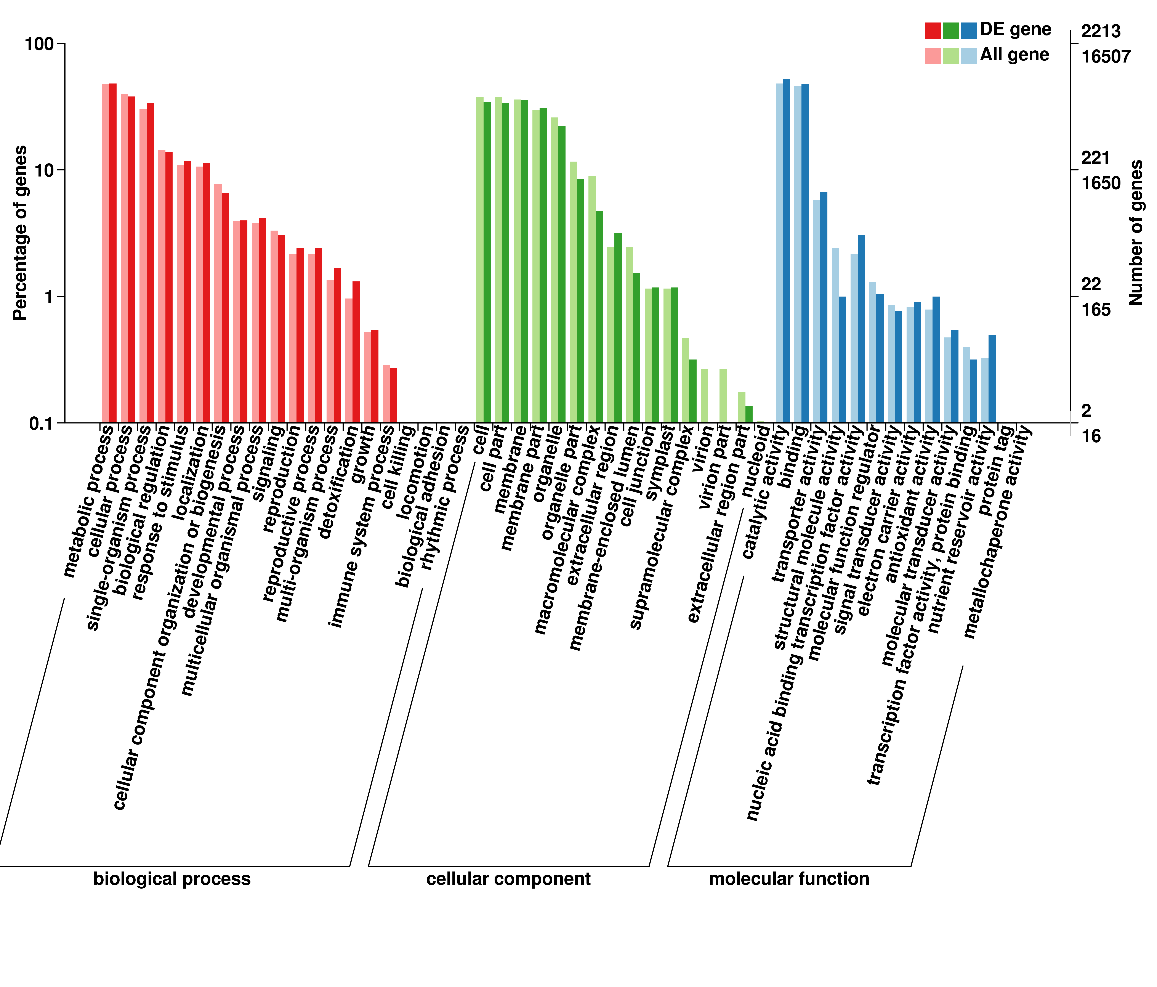


**Fig. S4.** GO classifications of DEGs at S0, S1 and S3.
