# Supplementary material for: Potential Association of Reactive Oxygen Species With Male Sterility in Peach
Source: Front Plant Sci. 2021 Apr 14;12:653256. doi: 10.3389/fpls.2021.653256 (PMC8079786; doi:10.3389/fpls.2021.653256)
Supplement: Supplementary Table 1 — The primers used in this article. [file Table_1.docx]

Table S1. The primers used in this study

| Gene ID | 5’-3’ Forward primer | 5’-3’ Reverse primer |
| --- | --- | --- |
| Prupe.6G091600 | TCCAACTGACACTGCCCTTC | GCTCAGAAAGCCTCTGGTGT |
| Prupe.6G242200 | GACAAACAAGAACCGCCACC | TACCCAATGTGTGTCCACCG |
| Prupe.2G004500 | ACTCAGCAGATAACGCACCC | TTTGAGGCATAACCCATGCCC |
| Prupe.3G178500 | ATGTGGTGAGGTCTGTGCTG | AATTGCCCAAACAGCTCGTC |
| Prupe.5G105100 | CATGTCCAGCCTTGGAGTCA | GAGAGCAAACGTACCCAGCA |
| Prupe.6G072400 | TCCAGCAGGCAAAAACGGTA | GTTTGAACAGATGCGCCTGG |
| Prupe.1G054900 | GTGTCAAGGTTCTGGAGGCA | ACACGAGATTTCCTCAGGCG |
| Prupe.1G055000 | TCTGGTGGACATTGCCTGTG | CAGCGTGCCTGGTTCTATCA |
| Prupe.4G146400 | GAAGGTGCTACGGTCAACCA | ACCGCCAAAGTAAGGCTTGT |
| Prupe.4G146800 | TCGAGGAAAATATGGGGCACAA | GACACGCTCTCCCGGCG |
| Prupe.4G147400 | CCACTCTTGCTCCAGTCGAA | ACTGAACCGCAATGAGGGAC |
| Prupe.5G099700 | TCAAGCATGTCGCCCAAGAT | ACACCGGTTCTGACAACCTC |

Table S2. DEGs involved in ROS-related pathways and their description

| Gene ID | Log_2_^（JX_S0/ZH_S0)^ | Log_2_^（JX_S1/ZH_S1)^ | Log_2_^（JX_S3/ZH_S3)^ | | Description |
| --- | --- | --- | --- | --- | --- |
| Prupe.1G111300.1 | -0.80909 | 4.890383 | | -0.17312 | Galactinol synthase 2 |
| Prupe.2G277900.1 | 0.238969 | 1.631708 | | -1.48046 | Beta-fructofuranosidase, soluble isoenzyme |
| Prupe.2G286900.1 | -0.53245 | 1.709079 | | -0.86348 | Phosphoglucomutase |
| Prupe.3G005100.1 | 1.656029 | -2.10219 | | -1.41823 | Galactinol synthase 1 |
| Prupe.3G289900.1 | 0.006749 | 1.638681 | | 0.495602 | Probable galactinol--sucrose galactosyltransferase 5 |
| Prupe.4G103500.1 | 0.292443 | 2.439978 | | -0.10526 | Alpha-glucosidase |
| Prupe.5G075600.1 | 0.420211 | 2.714608 | | -0.93262 | Acid beta-fructofuranosidase |
| Prupe.5G162400.1 | 0.186529 | 2.270483 | | #DIV/0! | Probable galactinol--sucrose galactosyltransferase 2 |
| Prupe.6G032400.1 | -0.51948 | 2.022509 | | -0.86687 | Probable galactinol--sucrose galactosyltransferase 1 |
| Prupe.6G309400.1 | 0.094398 | 1.535075 | | 0.006352 | Galactinol synthase 2 |
| Prupe.7G103100.1 | 3.254938 | 0.458866 | | -0.11381 | Beta-fructofuranosidase, insoluble isoenzyme 1 |
| Prupe.7G103200.1 | 8.034914 | -0.29167 | | 1.407276 | Beta-fructofuranosidase, insoluble isoenzyme 1 |
| Prupe.7G103400.1 | 2.991079 | -0.33911 | | -0.78042 | Beta-fructofuranosidase, insoluble isoenzyme 1 |
| Prupe.7G216700.1 | 1.061694 | 2.08694 | | -0.33596 | 6-phosphofructokinase 3 |
| Prupe.7G248600.1 | 0.226471 | -0.47132 | | -1.20549 | Probable galactinol--sucrose galactosyltransferase 6 |
| Prupe.1G079800.1 | 10.21961696 | -4.933669228 | | -1.747393075 | Spermidine hydroxycinnamoyl transferase |
| Prupe.1G227100.1 | 5.221395212 | 1.194366092 | | -0.509917558 | Flavonoid 3&amp;apos;,5&amp;apos;-methyltransferase |
| Prupe.1G305100.1 | -0.244869151 | 1.914027633 | | -0.335853151 | Leucoanthocyanidin reductase |
| Prupe.1G376400.1 | -0.730374681 | 2.858588959 | | -0.005714205 | Bifunctional dihydroflavonol 4-reductase/flavanone 4-reductase |
| Prupe.1G502800.1 | 6.264122358 | 0.840220628 | | 0.277143428 | Flavonol synthase/flavanone 3-hydroxylase |
| Prupe.1G580200.1 | 1.621157185 | 4.611444196 | | 0.512700079 | Cytochrome P450 |
| Prupe.1G580400.1 | -0.419237958 | 1.88538861 | | 0.22126249 | Cytochrome P450 |
| Table S2. (continued) | | | | | |
| Gene ID | Log_2_^（JX_S0/ZH_S0)^ | Log_2_^（JX_S1/ZH_S1)^ | | Log_2_^（JX_S3/ZH_S3)^ | Description |
| Prupe.1G580500.1 | 0.10106196 | -1.777097961 | | 0.062061914 | Cytochrome P450 |
| Prupe.2G107300.1 | 1.203774849 | -6.327085925 | | 0.813738234 | Flavonoid 3&amp;apos;,5&amp;apos;-methyltransferase |
| Prupe.3G101900.1 | 1.040404585 | 0.893241383 | | -0.080817189 | Shikimate O-hydroxycinnamoyltransferase |
| Prupe.4G029700.1 | -0.749293381 | -1.027246431 | | -0.042643069 | Anthocyanidin reductase |
| Prupe.7G168300.1 | 0.233063437 | 2.347190168 | | -0.956094605 | Naringenin,2-oxoglutarate 3-dioxygenase |
| Prupe.7G214300.1 | 12.03757546 | 1.611340411 | | -0.392083798 | Probable caffeoyl-CoA O-methyltransferase |
| Prupe.1G079800.1 | 11.66345487 | -4.933669228 | | -1.747393075 | Spermidine hydroxycinnamoyl transferase |
| Prupe.1G227100.1 | 5.221395212 | 1.194366092 | | -0.509917558 | Flavonoid 3&amp;apos;,5&amp;apos;-methyltransferase |
| Prupe.1G580200.1 | 1.621157185 | 4.611444196 | | 0.512700079 | Cytochrome P450 |
| Prupe.1G580400.1 | -0.419237958 | 1.88538861 | | 0.22126249 | Cytochrome P450 |
| Prupe.1G580500.1 | -0.10106196 | -1.777097961 | | 0.062061914 | Cytochrome P450 |
| Prupe.2G107300.1 | 1.203774849 | -6.327085925 | | 0.813738234 | Flavonoid 3&amp;apos;,5&amp;apos;-methyltransferase |
| Prupe.3G101900.1 | 1.040404585 | 0.893241383 | | -0.080817189 | Shikimate O-hydroxycinnamoyltransferase |
| Prupe.7G214300.1 | 12.03757546 | 1.611340411 | | -0.392083798 | Probable caffeoyl-CoA O-methyltransferase |
| Prupe.1G343000.1 | 0.765259 | -1.70696 | | 0.20424 | Inositol oxygenase 1 |
| Prupe.4G008700.1 | -0.85783 | 1.546085 | | -0.31661 | Inositol monophosphatase 3 |
| Prupe.5G106000.1 | -0.04229 | 2.135768 | | 0.180541 | Monodehydroascorbate reductase |
| Prupe.5G202800.1 | -2.72993 | 3.291445 | | -0.11937 | UDP-glucose 6-dehydrogenase 5 |
| Prupe.6G091600.1 | 0.100078 | -1.2966 | | 0.246074 | L-ascorbate peroxidase |
| Prupe.6G155100.1 | -1.40078 | 0.897907 | | -0.41 | L-ascorbate oxidase |
| Prupe.6G242200.1 | -0.34203 | -3.62723 | | -0.04133 | L-ascorbate peroxidase 2 |
| Prupe.7G046500.1 | 0.454284 | -1.01534 | | -0.11626 | L-galactono-1,4-lactone dehydrogenase |
| Table S2. (continued) |  |  | |  |  |
| Gene ID | Log_2_^（JX_S0/ZH_S0)^ | Log_2_^（JX_S1/ZH_S1)^ | | Log_2_^（JX_S3/ZH_S3)^ | Description |
| Prupe.7G046600.1 | 0.322728 | -1.28687 | | 0.01001 | L-galactono-1,4-lactone dehydrogenase |
| Prupe.7G216300.1 | 1.232736 | -3.98581 | | -0.29203 | Inositol oxygenase 4 |
| Prupe.2G004500.1 | 0.801803 | -3.52558 | | -0.85653 | Zeaxanthin epoxidase |
| Prupe.3G178500.1 | 0.772328 | -0.9941 | | -0.78699 | Phytoene synthase |
| Prupe.4G150100.1 | 1.629645 | 0.795348 | | 1.582127 | 9-cis-epoxycarotenoid dioxygenase NCED1 |
| Prupe.5G013100.1 | -1.73916 | 1.483952 | | -0.29312 | Abscisic acid 8&amp;apos;-hydroxylase 1 |
| Prupe.5G105100.1 | 1.927697 | 2.180293 | | 1.328164 | Beta-carotene 3-hydroxylase |
| Prupe.6G049800.1 | -0.0904 | -1.59562 | | 0.005704 | Beta-carotene hydroxylase 2 |
| Prupe.6G072400.1 | -1.96953 | -3.04343 | | 0.198279 | Abscisic acid 8&amp;apos;-hydroxylase 2 |
| Prupe.7G133800.1 | 1.074479 | 3.533392 | | 2.547025 | Abscisic acid 8&amp;apos;-hydroxylase 4 |
| Prupe.8G125800.1 | -0.38762 | 2.647007 | | 1.24015 | Abscisic acid 8&amp;apos;-hydroxylase 4 |
| Prupe.1G054800.1 | 1.024593862 | 5.223952351 | | 0.339238464 | Probable glutathione S-transferase |
| Prupe.1G054900.1 | -0.802739488 | -1.400041551 | | 0.92235463 | Glutathione transferase |
| Prupe.1G055000.1 | 0.667861309 | #NUM! | | 0.054623617 | Probable glutathione S-transferase |
| Prupe.3G013600.1 | 5.469973784 | 0.121145103 | | -0.435142234 | Glutathione S-transferase |
| Prupe.3G165500.1 | 0.257617704 | -1.015723604 | | 0.218393144 | Microsomal glutathione S-transferase 3 |
| Prupe.3G306600.1 | -1.778879476 | -0.74553951 | | 1.489056199 | Ribonucleoside-diphosphate reductase |
| Prupe.4G146000.1 | -0.642901818 | -3.04702482 | | -0.467609588 | Probable glutathione S-transferase |
| Prupe.4G146300.1 | 2.275100079 | #NUM! | | 0.171420441 | Probable glutathione S-transferase |
| Prupe.4G146400.1 | -1.252761974 | -6.053092035 | | 0.001733082 | Probable glutathione S-transferase |
| Prupe.4G146600.1 | -1.484657385 | -6.735590024 | | -0.311719335 | Probable glutathione S-transferase |
| Prupe.4G146800.1 | -0.431296993 | -3.915120291 | | 1.209089058 | Probable glutathione S-transferase |
| Table S2. (continued) |  |  | |  |  |
| Gene ID | Log_2_^（JX_S0/ZH_S0)^ | Log_2_^（JX_S1/ZH_S1)^ | | Log_2_^（JX_S3/ZH_S3)^ | Description |
| Prupe.4G147400.1 | -1.267999284 | -4.681930104 | | -0.149990608 | Probable glutathione S-transferase |
| Prupe.5G014900.1 | 2.309672367 | 1.464633448 | | 0.242630236 | Glutathione S-transferase |
| Prupe.5G191600.1 | -4.463575598 | #DIV/0! | | #DIV/0! | Glutathione S-transferase |
| Prupe.6G040700.1 | 0.121206475 | 2.987783875 | | 0.787271607 | Probable glutathione S-transferase |
| Prupe.6G091600.1 | 0.100077562 | -1.296597741 | | 0.246074395 | L-ascorbate peroxidase |
| Prupe.6G159900.1 | -1.248020154 | -0.739974081 | | 0.955435577 | Ribonucleoside-diphosphate reductase large subunit |
| Prupe.6G242200.1 | -0.342030575 | -3.627228106 | | -0.041329758 | L-ascorbate peroxidase 2 |
| Prupe.6G244700.1 | -0.266676683 | -1.792965517 | | 0.315335184 | Glutathione S-transferase |
| Prupe.8G044900.1 | 11.53089689 | -1.246046435 | | #NUM! | Ornithine decarboxylase |
| Prupe.8G210700.1 | -1.799243764 | -2.467809606 | | 0.564672084 | Glutathione transferase |
| Prupe.8G231700.1 | -0.083674261 | -0.984208554 | | -0.7769918 | Glutathione reductase |
| Prupe.1G107000.1 | -1.58097 | 1.573963 | | -0.26474 | S-adenosylmethionine synthase 2 |
| Prupe.1G334400.1 | 0.153437 | -2.85621 | | -0.17304 | Aspartate-semialdehyde dehydrogenase |
| Prupe.1G416900.1 | 3.683785 | -1.5046 | | -0.50965 | Branched-chain-amino-acid aminotransferase 3 |
| Prupe.1G451900.1 | 0.017182 | -2.35857 | | -0.14914 | Aspartate aminotransferase 3 |
| Prupe.1G490000.1 | -2.46542 | 2.478144 | | -0.38542 | 1-aminocyclopropane-1-carboxylate oxidase |
| Prupe.2G022600.1 | -5.07466 | -2.82705 | | 0.41728 | n/a |
| Prupe.2G172800.1 | -0.29176 | -1.03075 | | -0.14778 | L-3-cyanoalanine synthase 1 |
| Prupe.2G176900.1 | 3.417686 | 3.852436 | | 1.057138 | 1-aminocyclopropane-1-carboxylate synthase 1 |
| Prupe.3G133100.1 | 0.220999 | -2.05508 | | -0.18714 | Probable aminotransferase |
| Prupe.3G312900.1 | -0.28773 | 1.564174 | | -0.75048 | Aspartokinase 3 |
| Prupe.5G072700.1 | 0.0058 | -1.47382 | | 0.921862 | L-lactate dehydrogenase |
| Table S2. (continued) |  |  | |  |  |
| Gene ID | Log_2_^（JX_S0/ZH_S0)^ | Log_2_^（JX_S1/ZH_S1)^ | | Log_2_^（JX_S3/ZH_S3)^ | Description |
| Prupe.6G011600.1 | -1.21134 | 0.62071 | | 1.181245 | DNA (cytosine-5)-methyltransferase |
| Prupe.6G288600.1 | 0.398307 | 2.864717 | | 0.029138 | Alanine--glyoxylate aminotransferase 2 |
| Prupe.7G179500.1 | -0.27859 | 1.454208 | | -0.10875 | Serine acetyltransferase 4 |
| Prupe.7G212000.1 | -1.46169 | -1.7089 | | -0.42324 | 1-aminocyclopropane-1-carboxylate oxidase 1 |
| Prupe.8G159500.1 | -1.06962 | -5.0548 | | -0.61268 | methylthioadenosine/S-adenosylhomocysteine nucleosidase 1 |
| Prupe.3G052000.1 | 2.078690951 | -0.019843632 | | 0.060768159 | Inorganic pyrophosphatase 2 |
| Prupe.3G069500.1 | -0.329703927 | -0.743024855 | | -0.831752141 | Probable pyridoxal biosynthesis protein |
| Prupe.6G100100.1 | 0.468181005 | -0.997235571 | | 0.011144433 | Pyridoxal biosynthesis protein |
| Prupe.6G277500.1 | -0.001137568 | -0.856623411 | | -0.802159563 | Probable pyridoxal biosynthesis protein |
| Prupe.1G541200.1 | 0.02701 | -1.4744 | | -1.57365 | Phosphoenolpyruvate carboxykinase |
| Prupe.2G091600.1 | 0.095811 | -1.31156 | | 0.29384 | Malate dehydrogenase |
| Prupe.3G012200.1 | -0.6229 | -2.52578 | | 0.414941 | Protein DJ-1 homolog D |
| Prupe.3G012300.1 | 2.24291 | -0.9563 | | 0.002483 | Protein DJ-1 homolog D |
| Prupe.3G012900.1 | -0.86912 | -2.56557 | | 0.12872 | Protein DJ-1 homolog D |
| Prupe.4G259200.1 | 0.119715 | 1.509802 | | -0.37314 | Biotin carboxylase 1 |
| Prupe.5G072700.1 | 0.0058 | -1.47382 | | 0.921862 | L-lactate dehydrogenase A |
| Prupe.5G154600.1 | -1.60885 | 0.579243 | | 1.844943 | Malate synthase |
| Prupe.6G001600.1 | -0.33148 | -1.52444 | | -0.45475 | Pyruvate kinase |
| Prupe.7G120700.1 | -0.10681 | -2.00575 | | -0.54584 | Pyruvate kinase |
| Prupe.8G265300.1 | -0.5679 | 2.051814 | | -0.3203 | Fumarate hydratase 1 |
|  |  |  | |  |  |

Table S3. mitochondrial DEGs which involved in oxidative phosphorylation and their description

| Gene ID | Log_2_^（JX_S0/ZH_S0)^ | Log_2_^（JX_S1/ZH_S1)^ | Log_2_^（JX_S3/ZH_S3)^ | Description |
| --- | --- | --- | --- | --- |
| Prupe.1G061900.1 | 1.064726551 | 4.406141161 | -0.717266163 | Ubiquinol oxidase, mitochondrial |
| Prupe.1G421800.1 | -0.273650628 | -1.025144401 | -0.156260295 | NADH dehydrogenase [ubiquinone] iron-sulfur protein 4, mitochondrial |
| Prupe.3G231300.1 | -4.249691961 | 0.011177249 | 0.481701981 | External alternative NAD(P)H-ubiquinone oxidoreductase B2, mitochondrial |
| Prupe.4G037100.1 | 0.460406076 | 2.482542867 | 0.212376939 | External alternative NAD(P)H-ubiquinone oxidoreductase B3, mitochondrial |
| Prupe.4G244400.1 | 1.019969314 | -4.091075671 | -0.290365262 | ADP, ATP carrier protein, mitochondrial |
| Prupe.5G018700.1 | 0.208634138 | -3.299254633 | -1.320615264 | Ubiquinol oxidase 1a, mitochondrial |
